# Health sciences librarians’ instructional engagement in continuing education: a scoping review

**DOI:** 10.29173/jchla29656

**Published:** 2024-04-01

**Authors:** Jackie Phinney, Melissa Helwig, Melissa A. Rothfus, Kristy Hancock

**Affiliations:** 1 Instruction/Liaison Librarian: Dalhousie Medicine New Brunswick, Dalhousie University/Dalhousie Medicine New Brunswick, Saint John, NB; 2 Associate Dean Research & Scholarly Communications, Dalhousie University, WK Kellogg Health Sciences Library, Halifax, NS; 3 Scholarly Communications Librarian, Dalhousie University, WK Kellogg Health Sciences Library, Halifax, NS; 4 Evidence Synthesis Coordinator, Maritime SPOR SUPPORT Unit, Halifax, NS

## Abstract

**Objective:**

Healthcare professionals (HCPs) have an ongoing need for continuing education (CE) while Health Science Librarians (HSLs), accustomed to supporting a range of learning needs in a variety of contexts, are well situated to provide CE that addresses information retrieval, literacy, management, and more. To better understand the extent of HSL delivered CE activities, we undertook a scoping review to determine how HSLs instruct practicing HCPs in support of their CE.

**Methods:**

We searched for published and unpublished literature sources including PubMed (NCBI), Embase (Elsevier); Dissertations and Theses Global (ProQuest); CINAHL (EBSCO); Library, Information Science and Technology Abstracts (EBSCO); and Library Literature and Information Science Full Text (EBSCO). To identify unpublished sources, we searched the internet using Google and contacted two health sciences library listservs. We also performed backwards and forwards searching of our included sources.

**Results:**

Our database searches yielded 4842 sources, and we retrieved an additional 579 sources through supplementary retrieval methods. After duplicate removal and screening, we included 105 sources in this review. The included sources were published between 1970 to 2021 and covered a range of topics such as searching methods and tools, critical appraisal, and many more. Those related to evidence-based practice (EBP) appeared around 2001 and bibliometrics and bioinformatics arose after 2016. Publications depicting HSLs teaching CE most commonly occurred in academic settings. The most common population taught was nurses, followed by physicians. Most sources did not report using an information literacy framework or instructional design model, undertaking needs assessments, or reporting formal objectives or assessment.

**Conclusion:**

While HSLs are active supporters of EBP, we need to apply the same principles to our own professional practice. Formal structure of programming and program assessment combined with clear, detailed reporting can help to build a more robust evidence base to support future CE provision.

## Introduction

Healthcare professionals (HCPs) such as nurses, physicians, and their allied health colleagues undergo extensive training at the undergraduate and postgraduate levels to become competent in their professions, but this learning does not end once they obtain certification. Their need for life-long learning is formalized as continuing education (CE), also referred to as continuing professional development, faculty development (for clinicians holding faculty appointments with an educational program), and profession specific names such as continuing medical education (CME) and continuing nursing education (CNE) [1].

One driver of CE with clear implications for health science librarians (HSLs) is the ever-evolving tools for accessing biomedical literature, and the emergence of evidence-based practice/evidence-based medicine (EBP/EBM) which relies on them [2]. As seen throughout the literature, HSLs have long provided CE to HCPs, however gaps remain in our knowledge of how this is done and which HCPs are the primary beneficiaries. Swanburg et al.’s 2016 systematic review firmly situated HSLs in the EBP curriculum for students, noting that “EBP skills are closely tied to information literacy” [3], but the scope of their review did not include evidence on the use of information literacy (IL) frameworks in teaching. Such frameworks are highly relevant to understanding how librarians deliver purposeful instruction. Awareness and application of the Association of College & Research Libraries’ (ACRL) Information Literacy Framework in teaching delivered by HSLs has been explored [4], but a comprehensive synthesis of IL frameworks in librarian delivered CE is still missing from the literature. Notably, Swanburg et al.’s [3] review also excluded HCPs from consideration, thereby creating an opportunity for further evidence syntheses of HSL delivery of EBP instruction to HCPs within a CE context.

As part of the discussion surrounding purposeful instruction, Lauseng et al.’s recent scoping review of health informatics training conducted by libraries or librarians included practicing HCPs in their data, and within their conclusions they noted that the “paucity of included studies and activities demonstrates the need for more libraries to report on these educational activities, with sufficient details on the interventions and evaluation” [5]. While that review excluded evidence of teaching on “bioinformatics, data management or data science, literature/database searching, evidence-based practice, or general mobile apps” [5], other literature demonstrates that these topics are being taught by HSLs in a CE context [6–9]. There is also evidence that chronicles the more intricate nuances of HSL instruction to practicing HCPs, such as unconventional settings [10], a pilot project where measurable objectives were employed [11], or librarians’ considerations for how to ensure their teaching was suited to the online environment [12].

In addition to these illustrative examples of librarian delivered CE, there is compelling discussion that time constraints are a barrier to providing CE to HCPs [13], and that beyond the library “engaging learners can be especially challenging for continuing medical education (CME)” [14].

One way of increasing learner engagement is through accreditation, which allows instructors to formally evaluate their sessions “against defined standards by an external body for the purposes of quality assurance and enhancement” [15] and encourages participation in CME activities [16]. However, while there is evidence demonstrating the importance of HSLs to the accreditation process for CME [17], to our knowledge the prevalence of HSLs including accreditation as part of their own instructional planning for HCPs’ CE is still unknown.

Another factor in planning for librarian-delivered CE is the inclusion of needs assessments, which is vital in planning CPD activities and can be done formally or informally [18]. Patterson noted the importance of assessment in customizing hospital library experiences for users [19], and with varying levels of knowledge, interest, and technical capabilities between HCPs, conducting needs assessments aligns with the Universal Design for Learning framework which encourages instructors to “optimize relevance, value, and authenticity” for the learner [20]. HSLs have demonstrated their ability to conduct information needs assessments with practicing HCPs [21], yet reviewing the occurrence of needs assessments in the greater context of other instructional details is helpful for librarians’ own planning and development as instructors.

This scoping review proposes to contribute to the conversation on how HSLs are instructing HCPs in a CE context. It builds on others’ work by marrying together current gaps, such as which HCPs are most often taught CE by HSLs and which topics are included in this teaching. It will also consider where the delivery of HSL-led CE most commonly occurs, the adoption of IL frameworks or models, the use of accreditation standards in teaching, and the extent of assessment or evaluation of CE programming. This work will support the Medical Library Association’s sentiment that “librarians are educators” [22], and provide HSLs with useful evidence to benchmark their own teaching in support of HCPs CE.

## Methods

This scoping review is reported according to the Preferred Reporting Items for Systematic reviews and Meta-Analyses (PRISMA) for scoping reviews checklist [23] and the PRISMA for searching checklist [24]. A review protocol was developed prior to starting the review and was not published. Due to the nature of this project, research ethics approval was not required.

### Information sources & search strategy

Published and unpublished sources of evidence were considered for inclusion in this review. To identify published sources, six bibliographic databases were searched: PubMed (NCBI), Embase (Elsevier); Dissertations and Theses (ProQuest); Cumulative Index to Nursing and Allied Health Literature (CINAHL) Full Text (EBSCO); Library, Information Science and Technology Abstracts (LISTA; EBSCO); and Library Literature and Information Science Full Text (EBSCO) (see Online Supplement Appendix 1 for searches). The initial search strategy was developed by an HSL on our team in PubMed using both index terms and keywords to describe the following key concepts: librarians, HCPs, and instruction. The PubMed search was peer-reviewed by another health sciences librarian (see Acknowledgements) using the Peer Review of Electronic Search Strategies (PRESS) checklist [25] and revised according to the feedback received before being translated to the other databases.

Online Supplement Appendix

The database searches were run on March 12, 2020 with no language or other limits used, and again on September 21, 2021 with date limits applied to retrieve results from March 2020 onward. The results from the database searches were imported to Covidence systematic review software [26] and duplicate records were automatically removed with additional duplicates removed during screening.

To identify unpublished sources, we searched the internet using Google on March 16, 2022 (see Online Supplement Appendix 1 for searches) and reached out to two HSL listservs (MEDLIB-L and CANMEDLIB) on November 12, 2021, and again on December 2, 2021. Searching for unpublished literature at these junctures was intentional, as it allowed us to gain a better understanding of the topic through screening initial database results prior to seeking grey literature from broader sources.

### Eligibility criteria

Sources were included if they met the following criteria:

- Mention of a health sciences library or health research setting
- Detailed description of HSL involvement in delivering planned instruction to HCPs
- English language

For this scoping review, instruction was defined as the process of teaching skills related to finding, retrieving, analyzing or using information in bioscience, clinical and health settings. This definition was informed by the Medical Library Association (MLA) competency on instruction [22] and the American Library Association’s broader definition of IL [27]. We included both group and individual teaching encounters, and initiatives could be on any topic, in any format (e.g., in-person or online), and delivered across a variety of disciplines if the audience was practicing HCPs (i.e., nurses, physicians, pharmacists, physiotherapists, dentists) and/or health sciences faculty. All study designs were considered for inclusion. Only English language articles were considered for inclusion due to author language restrictions, but abstracts were provided by the databases in English and ultimately no non-English sources were advanced to full-text screening. We excluded primary teaching materials used by librarians and sources that described non-educational library services, such as literature searching, document retrieval, or repository development. We also excluded evidence that focused on traditional research consultations, individual reference questions, and librarians as co-collaborators on research teams, as our focus was on detailed reports of planned instruction. Although undergraduate and graduate-level trainees are important contributors to the healthcare system, we excluded evidence that reported on librarians training these groups exclusively to meet curricular requirements but included evidence where these groups attended training events for HCPs. In line with MLA’s Competency 3 which states that librarians “share our expertise with one another” [22], we included evidence where other librarians were also in attendance at a CE event primarily directed at HCPs that was being taught by a colleague, but excluded it if the librarians were the main target audience.

### Selection of sources

We screened published sources using Covidence and conducted pilot-testing at the start of each screening stage to ensure consistent understanding of the eligibility criteria. At the title/abstract and full-text levels, each record was independently screened by two reviewers and disagreements were resolved through consensus. We executed two search strings in Google and for each search string screened the first 100 hits directly on the results page. Due to Google’s variability in results by date of search, location, etc., two reviewers conducted this screening together during a virtual meeting, opening up relevant items in a new window and discussing their eligibility. The items retrieved from the listserv call were screened separately by two reviewers in duplicate. We also conducted backwards searching by scanning the reference lists of included sources, and forwards searching using Scopus (Elsevier) to capture references citing our included sources. Relevant sources identified through backwards and forwards searching were imported into Covidence and screened independently by two reviewers at both the title/abstract and full-text levels.

### Data extraction

We performed data extraction using Google Sheets [28] and pilot-tested the extraction form to ensure full understanding of the variables. Data from each included source were extracted by one reviewer and verified in full by a second reviewer. Pollock et al.[29] explain that the process of extracting data for scoping reviews can be iterative and may require a flexible approach, which could include adding additional variables throughout the extraction process. Therefore, we began with a standardized data extraction form but made collective decisions to revise the form and re-extract data points from all included studies as our understanding of the evidence base evolved. To simplify analysis for several variables, including “topics covered”, we coded using a data dictionary that we developed iteratively throughout the data extraction stage. Data extraction variables of interest included the background context of teaching initiatives such as learner population, details regarding the planning and delivery of teaching, use of evaluation modalities, teaching partnerships reported, and recommendations for future teaching and planning (see Online Supplement Appendix 2 for the list of variables we extracted).

### Synthesis of results

According to Pollock et al [29], scoping reviews sometimes require a more in-depth approach to analysis. For example, basic qualitative content analysis is “a descriptive approach to analysis and involves a process of open coding to allocate concepts or characteristics into overall categories” [29]. We followed a comparable approach for several data points (e.g., session type, topics covered, session format) by grouping similar data items into categories before recording frequency counts. For the target audience, rather than grouping similar roles into categories, we opted to synthesize the data based on the language used by authors when it was clearly provided. Qualitative data were extracted for the variable “Recommendations for future planning and teaching” in the form of direct quotations. We created tables to group the data into appropriate categories by area of interest (see Tables 1-3 and Online Supplement Appendix 3). The full data file is available here: https://doi.org/10.5683/SP3/SMX0Y2.

**Table 1:** Major characteristics of included sources

Characteristic	Details	Number of Sources
Year	1970-1985	n=4
	1986-2000	n=26
	2001-2015	n=51
	2016-2021	n=24
		
*Country	Canada	n=15
	USA	n=77
	Europe	n=11
	Other	n=3
		
Study Design	Program Description	n=83
	Quantitative	n=18
	Mixed Methods	n=4
		
**Library Type	Academic	n=62
	Hospital	n=50
	Special	n=3
	Other (Research Unit)	n=1

* Numbers do not add up to 105 due to one source reporting on a teaching event that took place in both Mexico and the United States

** Numbers do not add up to 105 due to some sources reporting multiple library types.

**Table 2:** Topics taught by publication date range

Year of Publication	Topics Covered
1970-1979	Free info resources (NLM), Information literacy,Publishing,PubMed,Searching methods/tools
1980-1989	Medline, Organization/Filing, Other databases, Searching methods/tools, Subject headings
1990-1999	Basic computer skills, CINAHL, Citation management, Critical appraisal, Emerging technology, EndNote, Free info resources (NLM), Grateful Med, Grey literature/websites, Information literacy, MEDLINE, Other databases, PubMed, Searching methods/tools
2000-2009	Basic computer skills, CINAHL, Citation Management, Cochrane Library, Critical appraisal, Current awareness tools, Data resources, Embase, EndNote, Evidence-Based Practice/Evidence-Based Medicine, Formulating questions, Full text access, Grateful Med, Greyliterature/websites, Information literacy, Library website, MEDLINE, MedlinePlus, Micromedex, Open Access, Other databases, Patient education, Point of care tools/mobile apps, Publishing, PubMed, Searching methods/tool, Selecting journal for publication, Statistics resources, Subject headings, Writing
2010-2019	Basic computer skills, Bibliometrics, Bioinformatics, Blogging, CINAHL, Citation management, Citing, Clinical procedures, Community Health/demographic data, Critical appraisal, Emerging technology, EndNote, Evidence-Based Practice/Evidence-Based Medicine, Formulating questions, Free info resources (NLM), Full text access, Grey literature/websites, Health literacy, Information literacy, Knowledge translation, Library website, MEDLINE, MedlinePlus, Online collaboration tools, Online public access catalogues, Open Access, Other databases, Patient education, Podcasts, Point-of-care tools/mobile apps, Presenting, Publishing, PubMed, RSS Feeds, Research data management, Scopus, Searching methods/tools, Selecting journals for publication, Social media,Statistics resources, Study designs, Subject headings, Teaching methods/skills, Web of Science, Wikis, Writing
2020-2021	Bibliometrics, Critical appraisal, Formulating questions, Free info resources (NLM), Grey literature/websites, Library website,MedlinePlus, Patient education, Presenting, Publishing, Research planning, Searching methods/tools, Selecting journal for publication, Writing

**Table 3:** Planning and delivery

Characteristic	Details	Number of Sources	Percentage	
Information Literacy Framework/ Instructional Design Model Used	Yes	n=6	6%	
	No	n=99	94%	
			
Session(s) Accredited	Yes	n=27	26%	
	No	n=78	74%	
			
Learning Objectives Reported	Yes	n=30	29%	
	No	n=75	71%	
			
Needs Assessment Reported	Yes	n=29	28%	
	No	n=76	72%	
			
Delivery Method	In-Person	n=86	82%	
	Online	n=8	8%	
	Mixed	n=10	10%	
	Unclear	n=1	1%	
			
*Session Type	Class/workshop	n=90	86%	
	Clinical unit visit	n=9	9%	
	Exhibit	n=2	2%	
	Individual consultation	n=9	9%	
	Mentorship	n=8	8%	
	Game	n=1	1%	
	Symposium	n=1	1%	
	Theatrical play	n=1	1%	
	Unclear	n=4	4%	
			
*Session Format	One-Shot	n=14	13%
	One-Shot (Recurring)	n=51	49%
	Course (Recurring)	n=12	11%
	Series	n=10	10%
	Series (Recurring)	n=7	7%
			
Hands-on Learning	Yes	n=72	69%
	No	n=33	31%
			
Evaluation Reported	Yes	n=86	82%
	No	n=19	18%

* Numbers do not add up to 105 due to some sources being classified as multiple session types and session formats

## Results

**Fig. 1:**
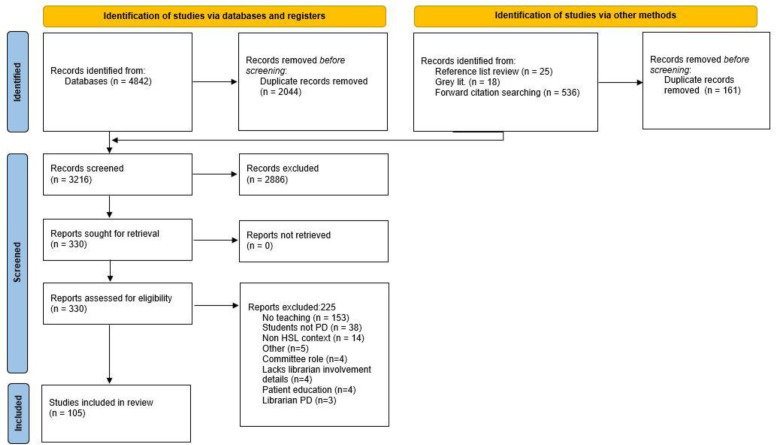
PRISMA flow diagram (adapted from Page MJ, McKenzie JE, Bossuyt PM, Boutron I, Hoffmann TC, Mulrow CD, et al. The PRISMA 2020 statement: an updated guideline for reporting systematic reviews. BMJ 2021 Mar 29;372:n71.

We identified 4842 records from our database searches, and 579 additional records through Google searching, listserv outreach, and backwards and forwards searching of included sources. After 2205 duplicates were removed, we screened 3216 records at the title/abstract level, assessed 330 reports for eligibility at the full-text level, and included 105 sources in this scoping review (see Figure 1 and Online Supplement Appendix 4 for the reference list of all included sources).

The most frequent publication date range of included sources was 2010-2019 (n=41, 39%), and the U.S. was the most common country of publication (n=77, 73%), with Canada being the second most common country (n=15, 14%). Most sources depicted teaching in an academic context (n=62, 59%) with hospital libraries being a close second (n=50, 48%). Within the hospital library data, over half of these sources (n=33, 66%) were attributed to American institutions whereas only 8 sources (16%) took place in the Canadian context. Special libraries (n=3, 3%) and research units (n=1, 1%) were represented far less. The majority of included sources were classified as a program description (n=83, 79%) (see Table 1 for major source characteristics).

The target population for sessions was most commonly nurses (n=57, 54%), followed by physicians (n=36, 34%), various health professionals (n=19, 18%), faculty (n=14, 13%), among others. Some sources (n=5, 5%) included librarians in attendance as well as HCPs (see Online Supplement Appendix 3 Table 1 for all populations reported).

A wide variety of topics were taught, and most included sources reported teaching more than one topic and were coded to reflect this. The most frequent topic taught was searching methods and tools (n=64, 61%), which we used as a code when authors were not more specific about the searching content or tools taught. Other topics included PubMed (n=25, 24%), MEDLINE (n=22, 21%), and grey literature/websites (n=21, 20%). A complete list of topics taught can be found in the full data file here: https://doi.org/10.5683/SP3/SMX0Y2.

Looking at topics taught by publication date range, PubMed was being taught as early as 1977 [30], and while it has been consistently taught in decades since, EBP/EBM and open access topics emerge in the 2000-2009 period, bibliometrics and bioinformatics begin being reported on in the 2010-2019 time period, and grey literature/websites emerge as a reported topic taught in the 1990-1999 time period (see Table 2 for all topics taught by date range).

With regard to the planning and delivery of sessions, the majority of sources (n=99, 94%) did not report using an IL framework or ID model, and 78 sources (74%) did not report accrediting their session (see Online Supplement Appendix 3 Table 2 for complete data on IL frameworks, ID models, and accrediting bodies reported). We also came across sources that described the influence of learning theories such as Kolb's experiential learning model [31,32] and Wilson's information behavior model [33] on teaching, but decided that learning theory fell outside of the scope of this review. Within the sources that reported using either an IL framework or ID model as part of the planning process, noted examples included different ACRL frameworks [34,35] and ADDIE [6,36] among others.

The use of needs assessments was low, with only 29 sources (28%) reporting the inclusion of this step in planning their instruction. Conversely, the majority of sources (n=86, 82%) included some sort of learner feedback, evaluation, or assessment as part of their teaching encounter. Most sources (n=75, 71%) did not explicitly report any learning objectives associated with their session(s). Turning to whether HSLs are including hands-on activities in their session, 72 sources (69%) reported hands-on learning as a component of their teaching (see Table 3 for all components of planning and delivery).

The details surrounding delivery of teaching revealed that the number of attendees was wide-ranging and uncertain, with 1-25 attendees (n=22, 21%) along with 100+ (n=22, 21%) being the most commonly reported ranges we calculated based on provided details. Aside from this, 25 included sources were unclear on their total number of attendees (24%). Most of our captured data depicted in-person teaching (n=86, 82%), with a much smaller number of sources describing a mix of in-person and online (n=10, 10%) or online only (n=8, 8%). One source (1%) was unclear about the delivery method for the session described. Data captured on the number of sessions taught were somewhat unclear as well, with 42 included sources (40%) not providing this information and the others ranging from 1 to 700 sessions discussed in a single paper [37] (See all captured data on this at the file found here: https://doi.org/10.5683/SP3/SMX0Y2).

In terms of session type, most included sources reported on librarians delivering a structured class or workshop session (n=90, 86%), but several unique examples described librarians developing and leading games [38], writing and presenting theatrical plays [39], and holding instructional exhibits [40,41]. The setting and formats of librarian teaching was fluid, as some sources described librarians visiting clinical teaching units to reach their audience [11,42–49], Some sources reported multiple session types and were coded as such. The most frequent session format was the recurring one-shot (i.e., a single session that was repeated exactly as run, multiple times), with 51 sources (49%) using this format far more than others and some sources reporting more than one format if multiple sessions took place.

On teaching partnerships, over half of the included sources (n=62, 59%) reported partnering with various professionals, faculty, professional organizations, hospital departments, and more in pursuit of the planning and delivery of their teaching endeavors (see Online Supplement Appendix 3 Table 3 for all partnerships reported). Authors also reported evidence of taking on additional tasks to supplement their instruction and support their learners. While 40 sources (38%) were unclear on the completion of additional teaching tasks, there were many accounts of HSLs providing independent learning materials (n=36, 34%), creating online resources (n=14; 13%), engaging in follow-up communications (n=14, 13%), and more (see Online Supplement Appendix 3 Table 4 for additional tasks done).

Finally, authors of 59 included sources (56%) provided recommendations for future planning and teaching. Some suggested adding motivation to participate through formal accreditation: “CME credit also helped establish the relevance of information management to clinical practice” [37]; the value in leveraging partnerships: “Establishing a collaborative relationship with nurses and other health professionals will boost the visibility of librarians and the stature and professional impact in EB processes” [43]; and realizing the continuing education potential of existing resources: “Many health sciences libraries already have educational programs in place on various topics...CE professionals can tap into this existing pool of courses and request that they be tailored to fit specific audiences at CE conferences or exhibits” [40]. For the full list of captured suggestions, see Online Supplement Appendix 3 Table 5.

## Discussion

Our scoping review sought to capture the specific details surrounding HSL teaching encounters in a CE context. Evidence from multiple countries, settings, and across different time periods was captured as part of this review, and our data show evidence that HSLs are actively contributing to the conceptualization, execution, and reflection of CE initiatives. While many of our data points warrant in-depth discussion and further research, the following major themes emerged during our analysis:

### Challenges with supporting further research

During the data extraction phase of this review, we found that the extracted data presented a number of challenges, including extensive gaps. One challenge is the language evolution observed over the course of time. For example, language evolution was captured from one source from 1974 that uses the term “allied health personnel” to describe workshop attendees which included administrators, secretaries, and nurses [50] who are now considered to be standalone professions that fall outside the allied health definition [51]. Such variation points to changes in context and possible limitations of the value of some older examples of CE.

A broader issue observed in the included studies was that details surrounding instructional activities were often limited. Most sources did not report learning objectives, the reported number of attendees and sessions was not always summarized clearly, and the language surrounding assessment methodologies was not always consistent (i.e., learner feedback, evaluation, and assessment were often used interchangeably). These gaps observed in examination of the literature can make it difficult for readers to reproduce the instructional activities described or reflect on the authors’ experiences. This raises the questions of the purpose of publishing on these experiences and why these elements of CE instruction have gone unaddressed. Further study to answer these questions is needed in order to address them.

### IL frameworks, instructional design (ID) models, and accreditation

The data from this scoping review demonstrate that HSLs are not always using IL frameworks or ID models in their teaching to practicing HCPs. Although very few included sources reported use of these tools, most of the sources were in the academic library context. This is consistent with the findings of Schulte and Knapp who noted that their study participants’ from academic settings were more likely to be aware of and engage with the ACRL Framework, and that “some librarians feel the framework is not relevant to their typical instructional settings or to the audiences to whom they typically provide instruction” [4].

Our data indicate that HSLs teaching CE sessions are largely forgoing the accreditation process. By doing so, HSLs may be limiting the appeal of their sessions for busy HCPs who need to regularly obtain CE credits as part of their roles. Future research could explore HSLs’ understanding of the CE accreditation process and willingness to engage with it.

### Setting

The data captured from our included studies show differences between academic and hospital librarians in providing CE to practicing health professionals with most of the teaching occurring in an academic setting. As the U.S. was the setting of many of these studies, future research might investigate whether differences between institutional staffing and structures, healthcare models, or funding opportunities influence this finding.

### Topics covered

Our data reflected a gradual evolution in topics being taught between 1970-2021 (our date of last search), which provides further evidence that librarians are remaining adaptable in their provision of CE. When analyzing data in the 2000-2009 time period, we see confirmation that the EBP/EBM movement had taken hold and librarians were now instructing on this along with formulating questions, neither of which appeared in our data prior to 2003 (See Table 2 for list of all topics taught by time period). We encourage future research into topics taught by HSLs to HCPs, as information-seeking behaviours evolve amidst new technologies and the fight against misinformation.

### Needs assessment

The data from this scoping review indicate that needs assessments are seldom conducted and attendees are not always being given a chance to voice their learning needs ahead of time. Future work could explore HSLs’ perceptions of needs assessments and if they feel they have the resources and time to engage with this important part of CE instruction.

### Librarians’ adaptability

The wide variety of settings, teaching formats, and participants in our data demonstrate that HSLs remain adaptable in their provision of CE to HCPs. This can be seen in the types of sessions that librarians are delivering, including unique examples such as games and theatrical plays. These examples of non-traditional instructional sessions in our findings also demonstrate that librarians are not afraid to think creatively in order to engage their audience. Librarians’ willingness to deliver instruction on clinical teaching units further demonstrates their motivation to adapt to the needs of busy HCPs, and one source discussed accepting opportunities to teach at CE events serendipitously as HCPs experienced their instructional offerings and invited them to attend more [10]. Librarians’ adaptability can also be found in the teaching partnerships reported in over half of the included sources. These partnerships can encourage alignment of educational offerings within continuing professional development programs, and further research could explore HSLs’ motivations for partnering with faculties in support of CE initiatives.

## Limitations

We recognize there were several limitations to this scoping review that could impact our results. While we conducted our data extraction and analysis in a manner supported by Pollock et al. [29], our method of grouping similar data items into overall categories was subject to our own biases and experiences as librarians.

We are also aware that reporting bias could have impacted the way that data were reported in our included sources, and that publication bias could have influenced librarians’ willingness to publish on their teaching experiences if they felt there was nothing worthwhile to communicate. Therefore, we recognize that our findings are not conclusive in nature and are merely drawn from what has been shared publicly by HSLs. We also acknowledge that our review could have been subject to language bias, as we searched English databases. Finally, the date of our last database search was September 2021, which makes this review somewhat dated. However, since that time we conducted an exploration of the grey literature, solicited evidence from stakeholders, and performed backwards and forwards searching, which all serve to bolster our methods.

## Conclusions

HSLs are well-positioned to provide CE to HCPs and should consider sharing fulsome accounts of their instructional endeavors and making use of robust methods of assessment so HSL pedagogy can continue to advance. The data from this scoping review indicate that the demands for content knowledge and expertise have evolved, according to what has been published in the literature up to September of 2021. At the same time, practices largely remain informal, with relatively few HSL-led CE sessions engaging in needs assessment, employing IL frameworks, articulating clear objectives, or accrediting their sessions. Future work should examine why this informal approach is taken, what impact it has on the success of CE sessions and whether development of recommended practices might add significant value to HSL led CE. By engaging in more formal structure for CE and program assessment, HSLs can develop a better evidence base to support future CE provision.

## Data Availability

All study characteristic data, along with the complete search strategies for this review and accompanying read me file, are available and can be downloaded from the institutional data repository found here: https://doi.org/10.5683/SP3/SMX0Y2.
