# Supplementary material for: Health sciences librarians’ instructional engagement in continuing education: a scoping review
Source: J Can Health Libr Assoc. 2024 Apr 1;45(1):30–43. doi: 10.29173/jchla29656 (PMC11081124; doi:10.29173/jchla29656)
Supplement: Supplementary file 1 — Online Supplement Appendix [file JCHLA-45-030-s001.pdf]

Online Supplement Appendix 1
Search Strategies

PubMed (NCBI):
Date searched: Inception-September 21, 2021

1. Librarians[Mesh] OR Library professional*[tiab] OR Library and Information professional*[tiab] OR Librarian*[tiab] OR information specialist*[tiab]
2. Health Personnel[Mesh] OR Health Occupations[Mesh] OR Health profession*[tiab] OR Health personnel[tiab] OR Nurses[Mesh] OR Nurse*[tiab] OR Nursing[tiab] OR Radiation Technolog*[tiab] OR Chiropract*[tiab] OR Laboratory Technolog*[tiab] OR Medical laboratory personnel[Mesh] OR Physiotherap*[tiab] OR Physical therapists[Mesh] OR Dietetic*[tiab] OR Nutritionist*[tiab] OR Nutritionists[Mesh] OR Dietitian*[tiab] OR Occupational therapists[Mesh] OR Occupational Therap*[tiab] OR Denturists[Mesh] OR Denturist*[tiab] OR Social workers[Mesh] OR Social Work*[tiab] OR Counselors[Mesh] OR Counselling Therapist*[tiab] OR Counsellor*[tiab] OR Optometrists[Mesh] OR Optometr*[tiab] OR Optician*[tiab] OR Respiratory Therap*[tiab] OR Dental[tiab] OR Dentists[Mesh] OR Dental hygienists[Mesh] OR Dentist[tiab] OR Dental Technicians[Mesh] OR Physicians[Mesh] OR Physician*[tiab] OR Surgeons[Mesh] OR Surgeon*[tiab] OR Doctor*[tiab] OR Psychiatrist*[tiab] OR Clinician*[tiab] OR Midwif*[tiab] OR midwives[tiab] OR Psychologist*[tiab] OR Pharmacy[tiab] OR Pharmacists[Mesh] OR Pharmacist*[tiab] OR Emergency Medical Technicians[Mesh] OR Paramedic*[tiab] OR Emergency Medical Technician*[tiab] OR "Speech-Language Pathology"[Mesh] OR speech language pathology*[tiab] OR Audiologists[Mesh] OR audiologist*[tiab] OR "Epidemiologists"[Mesh] OR epidemiologist*[tiab]
3. Education[Mesh] OR education[subheading] OR train*[tiab] OR educat*[tiab] OR workshop*[tiab] OR class*[tiab] OR course*[tiab] OR curriculum[tiab] OR instruct*[tiab] OR learn*[tiab] OR program*[tiab] OR teach*[tiab] OR taught[tiab] OR webinar*[tiab] OR faculty development*[tiab] OR professional development*[tiab] OR lunch and learn*[tiab] OR journal club*[tiab] OR information session*[tiab] OR competencies[tiab] OR brown bag lunch*[tiab]
4. 1 AND 2 AND 3

LISTA (Ebsco):
Date searched: Inception-September 21,2021

1. TI (Health profession* OR Health personnel OR Nurse* OR Nursing OR Radiation Technolog* OR Chiropract* OR Laboratory Technolog* OR Medical laboratory personnel OR Physiotherap* OR Physical therapist* OR Dietetic* OR Nutritionist* OR Dietitian* OR Occupational Therap* OR Denturist* OR Social Work* OR Counselor* OR Counselling Therapist* OR Counsellor* OR Optometr* OR Optician* OR Respiratory Therap* OR Dental OR Dentist* OR Physician* OR Surgeon* OR Doctor* OR Psych* OR Clinician* OR Midwif* OR midwives OR Psychologist* OR Pharmacist* OR Pharmacy OR Emergency Medical Technician* OR Paramedic* OR speech language patholog* OR audiologist* OR epidemiologist*) OR AB (Health profession* OR Health personnel OR Nurse* OR Nursing OR Radiation Technolog* OR Chiropract* OR Laboratory Technolog* OR Medical laboratory personnel OR Physiotherap* OR Physical therapist* OR Dietetic* OR Nutritionist* OR Dietitian* OR Occupational Therap* OR Denturist* OR Social Work* OR Counselors OR Counselling Therapist* OR Counsellor* OR Optometr* OR Optician* OR Respiratory Therap* OR Dental OR Dentist* OR Physician* OR Surgeon* OR Doctor* OR Psych* OR Clinician* OR Midwif* OR midwives OR Psychologist* OR Pharmacist* OR Pharmacy OR Emergency Medical Technician* OR Paramedic* OR speech language patholog* OR audiologist* OR epidemiologist*)
2. TI (Educat* OR train* OR workshop* OR class* OR course* OR curriculum OR instruct* OR learn* OR program* OR teach* OR taught OR webinar* OR faculty development* OR professional development* OR lunch and learn* OR journal club* OR information session* OR competencies OR brown bag lunch*) OR AB (Educat* OR train* OR workshop* OR class* OR course* OR curriculum OR instruct* OR learn* OR program* OR teach* OR taught OR webinar* OR faculty development* OR professional development* OR lunch and learn* OR journal club* OR information session* OR competencies OR brown bag lunch*)
3. TI ((hospital OR health OR medical OR nursing OR drug OR biomedical OR academic) N3 (Librar* OR information specialist)) OR AB ((hospital OR health OR medical OR nursing OR drug OR biomedical OR academic) N3 (Librar* OR information specialist))
4. 1 AND 2 AND 3

LLIS Full Text (Ebsco):
Date searched: Inception-September 21, 2021

1. TI (Health profession* OR Health personnel OR Nurse* OR Nursing OR Radiation Technolog* OR Chiropract* OR Laboratory Technolog* OR Medical laboratory personnel OR Physiotherap* OR Physical therapist* OR Dietetic* OR Nutritionist* OR Dietitian* OR Occupational Therap* OR Denturist* OR Social Work* OR Counselor* OR Counselling Therapist* OR Counsellor* OR Optometr* OR Optician* OR Respiratory Therap* OR Dental OR Dentist* OR Physician* OR Surgeon* OR Doctor* OR Psych* OR Clinician* OR Midwif* OR midwives OR Psychologist* OR Pharmacist* OR Pharmacy OR Emergency Medical Technician* OR Paramedic* OR speech language patholog* OR audiologist* OR epidemiologist*) OR AB (Health profession* OR Health personnel OR Nurse* OR Nursing OR Radiation Technolog* OR Chiropract* OR Laboratory Technolog* OR Medical laboratory personnel OR Physiotherap* OR Physical therapist* OR Dietetic* OR Nutritionist* OR Dietitian* OR Occupational Therap* OR Denturist* OR Social Work* OR Counselors OR Counselling Therapist* OR Counsellor* OR Optometr* OR Optician* OR Respiratory Therap* OR Dental OR Dentist* OR Physician* OR Surgeon* OR Doctor* OR Psych* OR Clinician* OR Midwif* OR midwives OR Psychologist* OR Pharmacist* OR Pharmacy OR Emergency Medical Technician* OR Paramedic* OR speech language patholog* OR audiologist* OR epidemiologist*)
2. TI (Educat* OR train* OR workshop* OR class* OR course* OR curriculum OR instruct* OR learn* OR program* OR teach* OR taught OR webinar* OR faculty development* OR professional development* OR lunch and learn* OR journal club* OR information session* OR competencies OR brown bag lunch*) OR AB (Educat* OR train* OR workshop* OR class* OR course* OR curriculum OR instruct* OR learn* OR program* OR teach* OR taught OR webinar* OR faculty development* OR professional development* OR lunch and learn* OR journal club* OR information session* OR competencies OR brown bag lunch*)
3. TI ((hospital OR health OR medical OR nursing OR drug OR biomedical OR academic) N3 (Librar* OR information specialist)) OR AB ((hospital OR health OR medical OR nursing OR drug OR biomedical OR academic) N3 (Librar* OR information specialist))
4. 1 AND 2 AND 3

Embase (Elsevier):
Date searched: Inception-September 21, 2021

1. ‘librarian’/exp OR “Library professional*”:ab,ti OR “Library and Information professional*”:ab,ti OR Librarian*:ab,ti OR “information specialist*”:ab,ti
2. ‘Health care personnel’/exp OR Health profession*:ab,ti OR Health personnel:ab,ti OR ‘Nurse’/exp OR Nurse*:ab,ti OR Nursing:ab,ti OR “Radiation Technolog*”:ab,ti OR Chiropract*:ab,ti OR “Laboratory Technolog*”:ab,ti OR ‘Clinical laboratory personnel’/exp OR Physiotherap*:ab,ti OR ‘Physiotherapist’/exp OR Dietetic*:ab,ti OR ‘Dietician’/exp OR Dietician*:ab,ti OR Nutritionist*:ab,ti ‘Occupational therapist’/exp OR “Occupational Therap*”:ab,ti OR ‘Denturist’/exp OR Denturist*:ab,ti OR ‘Social worker’/exp OR “Social Work*”:ab,ti OR ‘Counselor’/exp OR “Counselling Therapist*”:ab,ti OR Counsellor*:ab,ti OR ‘Optometrist’/exp OR Optometr*:ab,ti OR Optician*:ab,ti OR “Respiratory Therap*”:ab,ti OR Dental:ab,ti OR ‘Dentist’/exp OR ‘Dental hygienist’/exp OR Dentist:ab,ti OR ‘Dental Technician’/exp OR ‘Physician’/exp OR Physician*:ab,ti OR ‘Surgeon’/exp OR Surgeon*:ab,ti OR Doctor*:ab,ti OR Psychiatrist*:ab,ti OR Clinician*:ab,ti OR Midwif*:ab,ti OR Psychologist*:ab,ti OR Pharmacy:ab,ti OR ‘Pharmacist’/exp OR Pharmacist*:ab,ti OR ‘Rescue personnel’/exp OR Paramedic*:ab,ti OR “Emergency Medical Technician*”:ab,ti OR ‘Speech-Language Pathologist’/exp OR “speech language pathologist*”:ab,ti OR ‘Audiologist’/exp OR audiologist*:ab,ti OR ‘Epidemiologist’/exp OR epidemiologist*:ab,ti
3. ‘Education’/exp OR train*:ab,ti OR educat*:ab,ti OR workshop*:ab,ti OR class*:ab,ti OR course*:ab,ti OR curriculum:ab,ti OR instruct*:ab,ti OR learn*:ab,ti OR program*:ab,ti OR teach*:ab,ti OR taught:ab,ti OR webinar*:ab,ti OR “faculty development*”:ab,ti OR “professional development*”:ab,ti OR (lunch NEXT/2 learn*):ab,ti OR “journal club*”:ab,ti OR “information session*”:ab,ti OR competencies:ab,ti OR “brown bag lunch*”:ab,ti
4. 1 AND 2 AND 3

CINAHL (Ebsco):
Date searched: Inception-September 21, 2021

1. TI (Librarian* OR information specialist*) OR AB (Librarian* OR information specialist*)
2. TI (Health profession* OR Health personnel OR Nurse* OR Nursing OR Radiation Technolog* OR Chiropract* OR Laboratory Technolog* OR Medical laboratory personnel OR Physiotherap* OR Physical therapist* OR Dietetic* OR Nutritionist* OR Dietitian* OR Occupational Therap* OR Denturist* OR Social Work* OR Counselor* OR Counselling Therapist* OR Counsellor* OR Optometr* OR Optician* OR Respiratory Therap* OR Dental OR Dentist* OR Physician* OR Surgeon* OR Doctor* OR Psych* OR Clinician* OR Midwif* OR midwives OR Psychologist* OR Pharmacist* OR Pharmacy OR Emergency Medical Technician* OR Paramedic* OR speech language patholog* OR audiologist* OR epidemiologist*) OR AB (Health profession* OR Health personnel OR Nurse* OR Nursing OR Radiation Technolog* OR Chiropract* OR Laboratory Technolog* OR Medical laboratory personnel OR Physiotherap* OR Physical therapist* OR Dietetic* OR Nutritionist* OR Dietitian* OR Occupational Therap* OR Denturist* OR Social Work* OR Counselors OR Counselling Therapist* OR Counsellor* OR Optometr* OR Optician* OR Respiratory Therap* OR Dental OR Dentist* OR Physician* OR Surgeon* OR Doctor* OR Psych* OR Clinician* OR Midwif* OR midwives OR Psychologist* OR Pharmacist* OR Pharmacy OR Emergency Medical Technician* OR Paramedic* OR speech language patholog* OR audiologist* OR epidemiologist*) OR (MH “Health personnel”)
3. TI (Educat* OR train* OR workshop* OR class* OR course* OR curriculum OR instruct* OR learn* OR program* OR teach* OR taught OR webinar* OR faculty development* OR professional development* OR lunch and learn* OR journal club* OR information session* OR competencies OR brown bag lunch*) OR AB (Educat* OR train* OR workshop* OR class* OR course* OR curriculum OR instruct* OR learn* OR program* OR teach* OR taught OR webinar* OR faculty development* OR professional development* OR lunch and learn* OR journal club* OR information session* OR competencies OR brown bag lunch*)
4. 1 AND 2 AND 3

Dissertations and Theses (ProQuest):
Date searched: Inception-September 21, 2021

1. ti(Health profession* OR Health personnel OR Nurse* OR Nursing OR “Radiation Technolog*” OR Chiropract* OR “Laboratory Technolog*” OR “Medical laboratory personnel” OR Physiotherap* OR “Physical therapists” OR Dietetic* OR Nutritionist* OR Dietician* OR “Occupational therapists” OR “Occupational Therap*” OR Denturists OR Denturist* OR “Social workers” OR “Social Work*” OR Counselors OR “Counselling Therapist*” OR Counsellor* OR Optometrists OR Optometr* OR Optician* OR “Respiratory Therap*” OR Dental OR Dentists OR Physicians OR Surgeons OR Doctor* OR Psych* OR Clinician* OR Midwif* OR Midwives OR Psychologist* OR Pharmacist* OR Pharmacy OR “Emergency Medical Technician*” OR Paramedic* OR “speech language patholog*” OR audiologist* OR epidemiologist*) OR ab(Health profession* OR Health personnel OR Nurse* OR Nursing OR “Radiation Technolog*” OR Chiropract* OR “Laboratory Technolog*” OR “Medical laboratory personnel” OR Physiotherap* OR “Physical therapists” OR Dietetic* OR Nutritionist* OR Dietician* OR “Occupational therapists” OR “Occupational Therap*” OR Denturists OR Denturist* OR “Social workers” OR “Social Work*” OR Counselors OR “Counselling Therapist*” OR Counsellor* OR Optometrists OR Optometr* OR Optician* OR “Respiratory Therap*” OR Dental OR Dentists OR Physicians OR Surgeons OR Doctor* OR Psych* OR Clinician* OR Midwif* OR Midwives OR Psychologist* OR Pharmacist* OR Pharmacy OR “Emergency Medical Technician*” OR Paramedic* OR “speech language patholog*” OR audiologist* OR epidemiologist*)
2. ti(Educat* OR train* OR workshop* OR class* OR course* OR curriculum OR instruct* OR learn* OR program* OR teach* OR taught OR webinar* OR “faculty development*” OR “professional development*” OR lunch and learn* OR “journal club*” OR “information session*” OR competencies OR “brown bag lunch*”) OR ab(Educat* OR train* OR workshop* OR class* OR course* OR curriculum OR instruct* OR learn* OR program* OR teach* OR taught OR webinar* OR “faculty development*” OR “professional development*” OR “lunch and learn*” OR “journal club*” OR “information session*” OR competencies OR “brown bag lunch*")
3. ti((hospital OR health OR medical OR nursing OR drug OR biomedical OR academic) NEAR/3 (Librar* OR “information specialist”)) OR ab((hospital OR health OR medical OR nursing OR drug OR biomedical OR academic) NEAR/3 (Librar* OR “information specialist”))
4. 1 AND 2 AND 3

Google (Inception-March 16, 2022)

Search #1:
(librarian OR library) AND (“professional development” OR “continuing education”) AND (Health OR Biomedical) AND (clinician OR practitioner OR provider OR faculty) AND (filetype:pdf OR filetype:ppt)

Search #2:
(librarian OR library) AND (“professional development” OR “continuing education”) AND (Health OR Biomedical) AND (clinician OR practitioner OR provider OR faculty)

Online Supplement Appendix 2
Variables Extracted

The following data were extracted from the included papers:

- Publication Information
    - Title
    - Authors
    - Year
- Context
    - Source type
    - Study country
    - Library type
    - Study design
    - Accreditation reported (Y/N)
    - Accredited by whom
- Population
    - Target audience
- Education type
    - Session type
    - Topic/s covered
    - Learning objectives reported
    - Delivery (online, in-person, mix)
    - Hands on activities (Y/N)
    - IL Framework and/or Instructional design model reported (Y/N)
    - Framework and/or model used
    - Number of attendees reported (total)
    - Number of sessions/Programs/Events, etc.
    - Session format
    - Additional teaching tasks done
    - Teaching partnership (Y/N)
    - Partnership with
    - Evaluation or feedback reported
    - Needs assessment reported
- Recommendations
    - Recommendations for future planning and teaching

Online Supplement Appendix 3
Additional Data Tables

Table 1: Target Populations

Population & Number of Sources*
Nurses (n=57); Physicians (n=36); Various health professionals (n=19); Faculty (n=14); Staff (n=13); Administrators (n=10); Students (n=10); Public health personnel (n=9); Residents (n=7); Researchers (n=6); Clinicians (n=6); Physician assistants (n=5); Pharmacists (n=5); Librarians (n=5); Dentists (n=4); Dental hygienists (n=3); Educators (n=3); Epidemiologists (n=3); Nutritionists (n=3); Dieticians (n=3); Nurse residents (n=2); Physical therapists (n=2); Disease prevention specialists (n=2); Allied health professionals (n=2); Program directors (n=2); Social workers (n=2); Social service providers (n=2); Case managers (n=1); Patient educators(n=1); Hospital technicians (n=1); Hospital employees (n=1); Occupational therapists (n=1); Psychologists (n=1); Speech language pathologists (n=1); Recreational therapists (n=1); Rehabilitation assistants (n=1); Nurse practitioners (n=1); Medical records personnel (n=1); Community health professionals (n=1); Patient education committee members (n=1); Clerks (n=1); Research assistant (n=1); Physicians in training (n=1); Community members (n=1); Chiropractors (n=1); Veterinarians (n=1); Health planners (n=1); Outreach workers (n=1); Lab technicians (n=1); Environmental health specialists (n=1)

*Numbers do not equal 105 as sources often mentioned multiple target populations

Table 2: Information Literacy (IL) Frameworks/Instructional Design (ID) Models; Accrediting Bodies

IL Framework/ID Model & Number of Sources	Accrediting Body & Number of Sources
Analysis, Design, Development, Implementation, and Evaluation (ADDIE) Model (n=2); ACRL Framework (n=1); ACRL Information Literacy Competency Standards for Nursing (n=1); The Framework for Information and Communication Technology (ICT) (n=1); New York University Libraries' Information Literacy Competencies (n=1)	American Nurses Credentialing Center (ANCC) (n=3); American Medical Association (n=3); George Washington University Medical Center (n=2)*; Medical College of Ohio (n=2)*; Georgia Nurses Association (n=2); East Tennessee State University Office of Continuing Medical Education (n=2); American Medical Association/Florida Medical Association (n=1); College of Family Physicians of Canada; Royal College of Physicians and Surgeons of Canada (n=1); Croatian Chamber of Physicians (n=1); Glendale Adventist Medical Center (for nurses) (n=1); American Public Health Nurses Association (n=1)*; American Public Health Nurses Association(n=1)*; VA Boston Health Care System (n=1)*; New York University (n=1); Professional Organizations and CE departments at University of Utah (n=1); Rhode Island State Nurses Association (n=1)**; New Hampshire Nurses Association (n=1)**; New Hampshire Medical Society (n=1)**; Tennessee State Library Association (n=1); University of Arkansas Medical Center (n=1); University of Illinois College of Medicine (n=1); Virginia Commonwealth University School of Dentistry (n=1)

*Accreditation was implied by authors and therefore was captured as part of data extraction
**Different accrediting bodies mentioned in a single article

Table 3: Teaching Partnerships

Partnerships & Number of Sources*
Campus faculty (n=11); Health professionals (n=8); External organization(s) (n=7); Government department (n=6); CE coordinator/manager/office (n=5); Other librarians (n=4); Nursing research group (n=4); Nurse educators (n=3); Nursing leadership (n=3); Existing workshops (n=2); Professional association/center (n=2); Hospital department (n=2); Research scientists (n=1); Nurse scientist (n=1); Clinical practice sites (n=1); Health system project coordinator (n=1); Conference organizer (n=1); Not applicable (n=49)

*Numbers do not equal 105 as some sources mentioned multiple partnerships

Table 4: Additional Teaching Tasks

Additional Teaching Tasks & Number of Sources*
Provided independent learning materials (handout, articles, binder, chart, or course packet (n=36); Created online resource (n=14); Engaged in follow-up communications (n=14); Modified future teaching to meet user needs (n=1); Trained others for future teaching (n=1); Unclear (n=40)

*Numbers do not equal 105 as one paper mentioned multiple additional tasks done

Table 5: Recommendations for Future Planning and Teaching

Recommendations for Future Planning & Teaching (n=59 sources)
"Future offerings and follow-up should be more focused on the content identified in participants' plans for change, with less time spent on group discussions".(Allen et al., 2005)
"Other librarians should consider this format for their health care organizations if they are looking to produce an in-person and personalized, but uniform, EBP workshop".(Almader-Douglas et al., 2019)
"Include more regular meetings after the cohort have started writing at 6 months, and a 12-month touch base meeting to evaluate progress".<br><br>"Provid[e] more individual support by mentors prior to the first meeting".<br><br>"Celebrations to mark small successes in the writing process keep participants energized, whereas larger celebrations when manuscripts have been submitted/accepted will serve to recognize authors and inspire others to write".<br><br>"Completing a preworkshop self-assessment survey to measure knowledge gaps and comfort levels of the writer would be a valuable addition to the program". (Ansryan et al., 2019)
"This experience can be implemented across the board in any units in other areas of the hospital or in any hospital environment with the same or better results".<br>"An important part of the process is the need for librarians to study and understand evidence-based methodology, since EBP is not limited to health improvement".<br>"Establishing a collaborative relationship with nurses and other health professionals will boost the visibility of librarians and the stature and professional impact in EB processes".(Arguelles, 2011)
"Many health sciences libraries already have educational programs in place on various topics...CE professionals can tap into this existing pool of courses and request that they be tailored to fit specific audiences at CE conferences or exhibits".<br>"Librarians can assist in getting health professionals over the immediate barriers to access".(Arnold GN & Humphries AW, 1995)
"Evaluation of such a post within the NHS should focus on concrete benefits to the organization, such as whether the training is being used and for what".<br>"A balance of quantitative and qualitative methods can produce a well-rounded evaluation of such posts, adding narratives to usage rates".(Ayre, 2006)

Table 5: Recommendations for Future Planning and Teaching (Continued)

Recommendations for Future Planning & Teaching (n=59 sources)
"Support of the library's role in teaching information retrieval and management skills lies with full faculty endorsement".<br>"CME credit also helped establish the relevance of information management to clinical practice".<br>"No matter how good the program, people will not attend if it is not offered at a convenient time".<br>"Other considerations for a successful program are advertising, advanced registration, and follow-up confirmation".(Bader & Martin, 1988)
"Perhaps a hybrid model of several 'floating' librarians for groups to share would be the most cost-effective model where resources are limited".<br>"It was felt by librarians and participants alike, that ideally all rooms should have a networked PC or a laptop with access to a port".(Bexon N & Falzon L, 2003)
"The course could benefit from more interaction between participants and instructors. Rather than reviewing a critical appraisal checklist with the participants, for example, the group could work through the checklist with a sample article and discuss their results".<br>"Participants requested that more examples, hands-on materials, and discussion be included in the entire course".<br>"The test instrument would need to be revised so that questions are more rigorous".<br>"...focus on patient empowerment rather than patient education".<br>"Before teaching it again, the course's content and materials should be updated to reflect recent developments in EBP".(Blake & Ballance, 2013)
"A future survey to nursing contacts of needed educational content that can be provided by librarians will be administered allowing the development of new courses..."(Blake & Jaggers, 2020)

Table 5: Recommendations for Future Planning and Teaching (Continued)

Recommendations for Future Planning & Teaching (n=59 sources)
"The majority of our instructors felt that the time allotted to their presentation was too short and although the emphasis was on a common sense approach, we all agreed that there was no time allowed for practical work".<br>"We think now that eight weeks would be a better estimate of the time needed for proper instruction, including practical work".<br>"Separate courses with different emphases should be offered to each of these groups".(Borda, 1974)
"...the Medical Center's experience with other continuing medical education activities indicates that perhaps an afternoon schedule late in the week would have been preferable".(Bowen, 1977)
"...offer a periodic refresher training session".<br>"...replace the present three-hour lecture pus one-hour hands-on format with a format that alternates lecture and hands-on practice throughout the session".(Bredderman et al., 1988)
“Although relevant clinical information needs to remain the drawing card, when a short educational segment is embedded in related clinical information, health professionals are receptive to learning about information resources".(Burdick, 2011)
"An ideal expansion of the project would be to implement telemedicine services at the remote clinical practice sites".(Byrnes et al., 2004)
"Offering continuing education credit in medicine or other health sciences disciplines has increased participation in the past".<br>"One idea implemented is seeking co-sponsors and adding Appy Hour into a pre-existing event, symposium, or workshop. By collaborating with existing events, there is already a captive audience for Appy Hour. A future idea is adding a virtual connection option. This virtual connection could be either live streaming or a recording for viewing after Appy Hour".(Casucci et al., 2016)

Table 5: Recommendations for Future Planning and Teaching (Continued)

Recommendations for Future Planning & Teaching (n=59 sources)
"The pilot training sessions confirmed that one hour is inadequate to cover all aspects of EBP. When an hour or less is available, planning a series of training events is advisable".<br>"Many of the activities conducted as part of the Frontera Collaboration came about as a result of endorsements from leaders among clinicians and public health personnel in border communities".<br>"It may be difficult for clinicians and public health personnel to justify their attendance at training events. This challenge may be addressed by making the training easily accessible as part of regularly scheduled meetings or through web conferencing software".<br>"Providing continuing education credit may make a training session more appealing for clinicians and many public health personnel".<br>"In this context, it is important to highlight open-access publications and other freely available resources for evidence-based information".(Cogdill et al., 2012)
"Challenges facing all EBP projects and programs are: continued education in the concepts of change management, identification of change champions within the organizations, monitoring project relevancy, and ensuring project sustainability".(Coleman et al., 2018)
"Emphasize "coaching" rather than explaining as the teaching method".<br>"Word of mouth was the best advertisement".<br>"[Budget accordingly] so that software can be used immediately".<br>"Start and maintain a list of participants on a database..."(Craig JL et al., 1994)
"...librarians did not gain a good understanding of the week ahead until they met during the pre-workshop session...A short handbook is being created to address this issue..."<br>"...a major criticism expressed by participants and librarians was the lack of internet access in the small group meeting rooms".(Crumley E et al., 2002)
"nursing faculty members, whether on the tenure track or not, prefer synchronous online coursework to asynchronous coursework when they are learning about the process of writing for publication".(Dhakal & Tornwall, 2020)
"Educational efforts should concentrate not only on individual health practitioners but also on the institutions where they practice".(Dorsch, 1997)

Table 5: Recommendations for Future Planning and Teaching (Continued)

Recommendations for Future Planning & Teaching (n=59 sources)
"The presentation currently refers to EndNote as a useful tool in research and publication process, and the librarians may begin to offer training in the use of that software".<br>"...it's possible that this topic would work well on a Web-based platform".<br>"If sufficient interest is expressed among physicians, CME credit will be pursued for the course".(Doyle & Harvey, 2005)
"Faculty-Librarian partnerships are vital to the academic world". (Durando et al., 2007)
"...members of the health sciences librarianship profession could adapt this basic design of delaying an intervention for a control group".(Eldredge et al., 2008)
"If the program was redesigned today to incorporate some of these findings, library staff would try to put more social aspects back into the course".(Farrell et al., 2011)
"A follow-up survey of the participants was recommended to determine any increase in the library's electronic database use..."<br>"The majority thought that the game was fun and educational, but that the timeline for the game was too long".(Gage et al., 2011)
"When working with health professionals, librarians need to emphasize utilities for timesaving and clinical decision making, including empowering the health professionals to search the literature independently".<br>"Due to the busy schedules of the dental professionals, the librarians found that it was best to plan these sessions during regularly scheduled staff meetings at the clinics, with additional small group meetings planned as opportunities to follow up with participants".(Gaines et al., 2011)
"There was a strong desire that hands-on access be made available after each segment of the workshop".(Ginn & Tylman, 1987)
"Making the librarian-led sessions interactive by using online platforms such as Kahoot and Padlet greatly increased the reception, enthusiasm, and involvement..."(Glotfelty-Scheuering, 2019)
"Although most of the participants rated the opportunity for hands-on training as adequate, it is obvious that the ideal situation would be for each participant to have access to their own PC for all of the sessions".(Hartmann, 1998)

Table 5: Recommendations for Future Planning and Teaching (Continued)

Recommendations for Future Planning & Teaching (n=59 sources)
"Librarians/public health officials may benefit from interinstitutional collaboration and focused hands-on training..."<br>"Alternative strategies to increase staff participation in these trainings include making participation mandatory and expanding the number of times that these trainings are offered".(Harwell et al., 2008)
"The initial low rate of participation in training suggests that perhaps others in the physician's office, such as medical secretaries or medical assistants, might be appropriate primary targets for Internet training".(Hollander & Lanier, 1995)
"This case study illustrates the benefits of interdisciplinary collaboration between nurse educators and health sciences librarians to provide professional development opportunities".<br>"... the demographics and results may differ if the intervention was implemented in another geographic location".<br>"Those planning in-person PHN CE opportunities should be aware of any public health problems affecting their communities and maintain flexibility in regard to scheduling".(Johnson et al., 2017)
"Based on the pilot's success, the workshop will be offered again. Drawing on feedback from participants and instructors, more information on biomedical statistics and patient health literacy...will be included".<br>"Due to space constraints, the workshop served a small number of attendees. While a pre/post comparison was planned, there was an insufficient response from attendees, which limited ability to evaluate change in confidence to teach and practice EBP before and after the workshop".(Koffel & Reidt, 2015)

Table 5: Recommendations for Future Planning and Teaching (Continued)

Recommendations for Future Planning & Teaching (n=59 sources)
"...the team began the lesson planning with the goal of remaining flexible, always referring to the lesson plan as the “template” and intending it to be modified according to specific audience needs and interests".<br>"Networking can help identify new training audiences".<br>"...existing relationships... enabled the team to “hit the ground running,” knowing exactly who to contact for referrals to others potentially interested in the information the team aimed to impart".<br>"Assume nothing and learn as much as possible about a host’s or venue’s IT capabilities in advance of a presentation".<br>"Evaluations that include informed consent are more complicated in non-academic settings".<br>"... although team members were familiar with many of the groups in the training sessions, pre- and posttests or needs assessments could have been performed to identify exactly what HIV/AIDS information was known and what was needed".(Kuntz et al., 2018)
"...project will expand its focus to provide training on MedlinePlus and other consumer health resources to health care consumers".(Mani, 2008)
"In compiling the resources used in the training classes, all involved were reminded and once again impressed by the great wealth of high-quality research tools currently available, in great part thanks to the efforts of the National Library of Medicine (NLM). It was recognized, however, that even medical librarians contribute to the problem of their underuse by not promoting the freely available tools enough".(Matsoukas K., 2014)
"The reviews documented 2 consistently effective strategies: 1. performance audit with corrective, individualized feedback, and 2. preceptorships, that is, hands on experience under the supervision of an expert".(McKibbon et al., 1991)
"Methods of supporting nurses in CE by removing barriers should be a goal of future developers".<br>"Knowledge of the computer system participants will be using, increasing learner–learner and instructor–learner interactions, and creating easily navigated course content will all have positive effects on future participant satisfaction and completion rates".(Mears & Blake, 2017)

Online Supplement Appendix 4

References Included in this Scoping Review (n=105)

Aitken, E. M., Powelson, S. E., Reaume, R. D., & Ghali, W. A. (2011). Involving clinical librarians at the point of care: Results of a controlled intervention. *Academic Medicine : Journal of the Association of American Medical Colleges*, *86*(12), 1508–1512. https://doi.org/10.1097/ACM.0b013e31823595cd

Allen, M. P., Jacobs, S. K., Levy, J., Pierce, S., Pravikoff, D. S., & Tanner, A. (2005). Continuing education as a catalyst for inter-professional collaboration. *Medical Reference Services Quarterly*, *24*(3), 93–102. https://doi.org/10.1300/J115v24n03_08

Alligood, E. (2018). Nursing shared governance: Hospital librarians collaborating for quality. *Journal of Hospital Librarianship*, *18*(2), 160–167.

Almader-Douglas, D., Brigham, T., Marks, L., & Jett, H. (2019). Elevating evidence-based practice: A multi-site workshop pilot for nurses. *Medical Reference Services Quarterly*, *38*(4), 387–401. https://doi.org/10.1080/02763869.2019.1657737

Ansryan, L. Z., Marshall, C., Udin Aronow, H., Chan, A., & Coleman, B. (2019). Inspiring writing in nursing: A clinical nurse specialistYLed program. *Clinical Nurse Specialist: The Journal for Advanced Nursing Practice*, *33*(2), 90–96. https://doi.org/10.1097/NUR.0000000000000427

Antoniewicz, C. M. (1988). Teaching end users how to search the National Library of Medicine's toxicology databases. *Medical Reference Services Quarterly*, *7*(2), 31–39. https://doi.org/10.1300/J115v07n02_03

Arguelles, C. (2011). Evidence-based practice mentors: Taking information literacy to the units in a teaching hospital. *Journal of Hospital Librarianship*, *11*(1), 8–22.

Arnold, G., & Humphries, A. (1995). Continuing education and health sciences libraries: Opportunities for collaboration. *Journal of Continuing Education in the Health Professions*, *15*(4), 235–240.

Ayre, S. (2006). Workplace-based information skills outreach training to primary care staff. *Health Information & Libraries Journal*, *23 Suppl 1*, 50–54. https://doi.org/10.1111/j.1471-1842.2006.00676.x

Bader, S. A., & Martin, E. R. (1988). Teaching medical professionals to retrieve and manage medical information. *The Journal of Biocommunication*, *15*(2), 27–29.

Bader, S., & Martin, E. (1987). New methods of information management: New roles for the health sciences library. *Medical Reference Services Quarterly*, *6*(3), 63–68.

Bexon, N., & Falzon, L. (2003). Brief communication. Personal reflections on the role of librarians in the teaching of evidence-based healthcare. *Health Information & Libraries Journal*, *20*(2), 112–115.

Blake, L., & Ballance, D. (2013). Teaching evidence-based practice in the hospital and the library: Two different groups, one course. *Medical Reference Services Quarterly*, *32*(1), 100–110. https://doi.org/10.1080/02763869.2013.749143

Blake, L., & Jaggers, A. (2020). Touring Arkansas: A statewide project to instruct nurses and patient educators on consumer health resources. *Journal of Consumer Health on the Internet*, *24*(1), 64–74.

Borda, E. (1974). Introduction to library services for allied health personnel. *Bulletin of the Medical Library Association*, *62*(4), 363–366.

Boulos, L., McLean, K., Killian, L., Parker, R., & Helwig, M. (2018). *Lights, camera, learn! A cross-organizational approach to noon-hour information literacy instruction*. Canadian Health Library Association Annual Conference, St. John’s, Newfoundland and Labrador.

Bowen, M. (1977). On-line literature retrieval as a continuing medical education course. *Bulletin of the Medical Library Association*, *65*(3), 384–386.

Branden, S., & Wehmeyer, J. M. (1985). Do-it-yourself computer searching: Launching an educational program for the end user searcher. *Medical Reference Services Quarterly*, *4*, 11–14.

Bredderman, P. J., Wojcik, J., & Flake, D. (1988). BRS Colleague MEDLINE end user training follow-up survey. *Medical Reference Services Quarterly*, *7*(3), 15–34. https://doi.org/10.1300/J115v07n03_02

Burdick, A. J. (2011). Information “sound bites” by e-mail: Increasing information awareness and improving clinical information skills. *Journal of Hospital Librarianship*, *11*(1), 70–77.

Burrows, S. C., Moore, K. M., & Lemkau, H. L., Jr. (2001). Creating a web-accessible, point-of-care, team-based information system (PoinTIS): The librarian as publisher. *Bulletin of the Medical Library Association*, *89*(2), 154–164.

Byrnes, J. A., Kulick, T. A., & Schwartz, D. G. (2004). Information-seeking behavior changes in community-based teaching practices. *Journal of the Medical Library Association*, *92*(3), 334–340.

Casucci, T., Gregory, J. M., & Shipman, J. P. (2016). Appy hour: Health sciences professionals learn about apps. *Medical Reference Services Quarterly*, *35*(3), 251–258.

Cogdill, K. W., Ambriz, L., Billman, B. L., Carter, K. V., Nail-Chiwetalu, B., Trumble, J. M., El-Khayat, Y. M., & Nunez, A. V. (2012). The Frontera Collaboration: A preliminary report of health sciences librarians promoting evidence-based practice in U.S.-Mexico border communities. *Medical Reference Services Quarterly*, *31*(4), 400–413. https://doi.org/10.1080/02763869.2012.724285

Coleman, D. E., Kamai, S., & Davis, K. F. (2018). Impact of a collaborative evidence-based practice nursing education program on clinical operations. *Journal of Hospital Librarianship*, *18*(4), 323–330.

Collinge, B. (2006). Proving your worth as a clinical librarian. *Library & Information Update*, *5*(4), 36–37.

Craig, J., Gannon, P., & Brown, B. (1992). Providing individualized CME to geographically isolated specialists: A feasibility study. *Journal of Continuing Education in the Health Professions*, *12*(1), 15–23.

Craig, J. L., Kuruscz, S., & Gannon, P. (1994). Visiting librarian program. *Journal of Continuing Education in the Health Professions*, *14*(4), 232–238.

Crumley, E., Bayley, L., & Bhatnagar, N. (2002). Librarian integration into McMaster's 2001 evidence-based clinical practice workshop. *Bibliotheca Medica Canadiana*, *23*(3), 87–89.

Curtis, K. L., Weller, A. C., & Hurd, J. M. (1997). Information-seeking behavior of health sciences faculty: The impact of new information technologies. *Bulletin of the Medical Library Association*, *85*(4), 402–410.

Demczuk, L., Baker, A. C., Shaw-Daigle, C., & Raynard, M. (2006). Direct to you: Innovative information services to support nurses' continuing competence in Manitoba. *Journal of the Canadian Health Libraries Association (JCHLA)*, *27*(4), 109–111.

Dhakal, K., & Tornwall, J. (2020). The Scholarship Circle: An introduction to writing for publication for nursing faculty. *Journal of the Medical Library Association*, *108*(1), 98–105. https://doi.org/10.5195/jmla.2020.685

Dorsch, J. L. (1997). Equalizing rural health professionals' information access: Lessons from a follow-up outreach project. *Bulletin of the Medical Library Association*, *85*(1), 39–44.

Dorsch, J. L., & Landwirth, T. K. (1993). Rural GRATEFUL MED outreach: Project results, impact, and future needs. *Bulletin of the Medical Library Association*, *81*(4), 377–382.

Dorsch, J. L., Jacobson, S., & Scherrer, C. S. (2003). Teaching EBM teachers: A team approach. *Medical Reference Services Quarterly*, *22*(2), 107–114. https://doi.org/10.1300/J115v22n02_10

Doyle, J. D., & Harvey, S. A. (2005). Teaching the publishing process to researchers and other potential authors in a hospital system. *Journal of Hospital Librarianship*, *5*(1), 63–70.

Durando, P., Griffith, G., Halliday, S., Maranda, S., Ross-White, A., Smithers, A., Thomas, M., & Wickett, S. (2007). *Librarian and Faculty Partnerships: Teaching, Technology and Triumphs*. https://qspace.library.queensu.ca/bitstream/handle/1974/423/Durando%20P%20et%20al%20-%20Librarian%20and%20Faculty%20partnerships%20-%20Teaching%2C%20Technology%20and%20Triumphs%20-%20CHLA%202007%20poster.pdf?sequence=1&isAllowed=y

Eldredge, J. D., Carr, R., Broudy, D., & Voorhees, R. E. (2008). The effect of training on question formulation among public health practitioners: Results from a randomized controlled trial. *Journal of the Medical Library Association*, *96*(4), 299–309.

Eldredge, J. D., & Carr, R. D. (2007). Public health informatics training in New Mexico. *Journal of the Medical Library Association*, *95*(3), 343–346.

Farrell, A. M., Mayer, S. H., & Rethlefsen, M. L. (2011). Teaching Web 2.0 beyond the library: Adventures in social media, the class. *Medical Reference Services Quarterly*, *30*(3), 233–244. https://doi.org/10.1080/02763869.2011.590413

Gage, M., Peckman, G. S., & Greene, M. T. (2011). Using a “Survivor” style game to guide nursing research into practice. *Journal of Hospital Librarianship*, *11*(1), 94–98.

Gaines, J. K., Levy, L. S., & Cogdill, K. W. (2011). Sharing MedlinePlus®/MEDLINE® for information literacy education (SMILE): A dental public health information project.

Medical Reference Services Quarterly, *30*(4), 357–364. https://doi.org/10.1080/02763869.2011.609016

Giles-Smith, L., Spencer, A., Shaw, C., Porter, C., & Lobchuk, M. (2017). A study of the impact of an educational intervention on nurse attitudes and behaviours toward mobile device and application use in hospital settings. *Journal of the Canadian Health Libraries Association (JCHLA)*, *38*(1), 12–29.

Ginn, D. S., & Tylman, W. T. (1987). Evolution of an end-user training program. *Bulletin of the Medical Library Association*, *75*(2), 117–121.

Glotfelty-Scheuering, O. A. (2019). Leveraging the librarian in a nurse residency program. *Medical Reference Services Quarterly*, *38*(2), 113–130. https://doi.org/10.1080/02763869.2019.1588043

Grabeel, K. L., & Beeler, C. J. (2018). Taking the pulse of the University of Tennessee Medical Center’s health literacy knowledge. *Medical Reference Services Quarterly*, *37*(1), 89–96. https://doi.org/10.1080/02763869.2017.1404399

Gregg, A. L., & Wozar, J. A. (2003). Delivering Internet health resources to an underserved health care profession: School nurses. *Journal of the Medical Library Association*, *91*(4), 398–403.

Hartmann, J. (1998). An educational program in the medical uses of computers for rural physicians. *Medical Reference Services Quarterly*, *17*(3), 25–34. https://doi.org/10.1300/J115v17n03_03

Harwell, T. S., Law, D. G., Ander, J. L., & Helgerson, S. D. (2008). Increasing state public health professionals’ proficiency in using PubMed. *Journal of the Medical Library Association*, *96*(2), 134–137. https://doi.org/10.3163/1536-5050.96.2.134

Hayes, S., & Davis, B. B. (2014). The use of the play format for education in hospital environments: A case series. *Journal of Hospital Librarianship*, *14*(1), 41–51. https://doi.org/10.1080/15323269.2014.859996

Haynes, R. B., Johnston, M. E., McKibbon, K. A., Walker, C. J., & Willan, A. R. (1993). A program to enhance clinical use of MEDLINE. A randomized controlled trial. *The Online Journal of Current Clinical Trials*, *Doc No 56*, [4005-words; 39 paragraphs].

Hollander, S. M., & Lanier, D. (1995). Orientation to the Internet for primary care health professionals. *Bulletin of the Medical Library Association*, *83*(1), 96–98.

Hook, S. A. (1996). Learning to use the Internet: An interactive team-taught workshop for dental faculty and staff. *Bulletin of the Medical Library Association*, *84*(3), 409–411.

Hurt, E., & McLoughlin, A. (2021). Facilitating research amongst radiographers through information literacy workshops. *Journal of the Medical Library Association*, *109*(1), 112–119. https://doi.org/10.5195/jmla.2021.842

Johnson, E. M., Jones, K., Eathington, P., Howard, C., Raszewski, R., & Twigg, N. M. (2017). NExT: Creating an interdisciplinary alliance to diminish informational barriers for public health nursing. *Health Information & Libraries Journal*, *34*(3), 236–246. https://doi.org/10.1111/hir.12184

Koffel, J., & Reidt, S. (2015). An interprofessional train-the-trainer evidence-based practice workshop: Design and evaluation. *Journal of Interprofessional Care*, *29*(4), 367–369. https://doi.org/10.3109/13561820.2014.962127

Krom, Z. R., Batten, J., & Bautista, C. (2010). A unique collaborative nursing evidence-based practice initiative using the Iowa model: A clinical nurse specialist, a health science librarian, and a staff nurse’s success story. *Clinical Nurse Specialist*, *24*(2), 54–59. https://doi.org/10.1097/NUR.0b013e3181cf5537

Kuntz, G. M., Schaefer, N., Norton, H. F., & Tennant, M. R. (2018). HIV/AIDS outreach: Curriculum development and skills training to health and information professionals. *Medical Reference Services Quarterly*, *37*(1), 60–73. https://doi.org/10.1080/02763869.2018.1404389

Ladd, D. L., & Brody, E. R. (2020). Consumer oral health information resources workshop for dental faculty: A program overview and list of reliable online oral health information resources for consumers. *Journal of Consumer Health on the Internet*, *24*(1), 10–19.

Laibhen-Parkes, N. (2014). Increasing the practice of questioning among pediatric nurses: “The Growing Culture of Clinical Inquiry” project. *J Pediatr Nurs*, *29*(2), 132–142. https://doi.org/10.1016/j.pedn.2013.10.003

Lawton, A., Manning, P., & Lawler, F. (2017). Delivering information skills training at a health professionals continuing professional development conference: An evaluation. *Health Information & Libraries Journal*, *34*(1), 95–101.

Macdonald, K. (2015). Collaborative partnerships for library services: Examples from a hospital library. *Journal of Hospital Librarianship*, *15*(4), 365–372.

Maina, W. (1975). A class in library use for allied health personnel. *Bulletin of the Medical Library Association*, *63*(2), 226–228.

Mani, N. S. (2008). “Library-On-The-Go”: Utilizing technology to provide educational programming. *Journal of the Medical Library Association*, *96*(3), 230–232. https://doi.org/10.3163/1536-5050.96.3.009

Matsoukas, K. (2014). Assessing the health information needs of unaffiliated health professionals and using training on openly available search tools and resources to provide solutions to their information access challenges and barriers. *Medical Reference Services Quarterly*, *33*(4), 391–407. https://doi.org/10.1080/02763869.2014.957077

McCloskey, K. M. (2000). Library outreach: Addressing Utah’s “Digital Divide”. *Bulletin of the Medical Library Association*, *88*(4), 367–373.

McKibbon, K. A., Haynes, R. B., Johnston, M. E., & Walker, C. J. (1991). A study to enhance clinical end-user MEDLINE search skills: Design and baseline findings.

Proceedings of the Annual Symposium on Computer Application in Medical Care, 73–77.

Mears, K., & Blake, L. (2017). Nursing librarians cultivating evidence-based practice through an asynchronous online course. *The Journal of Continuing Education in Nursing*, *48*(9), 420–424. https://doi.org/10.3928/00220124-20170816-08

Mi, M. (2006). Evaluation of a hospital medical library class for NICU nurses. *Medical Reference Services Quarterly*, *25*(4), 21–35.

Miglus, J. D., & Froman, R. D. (2016). Evaluation of an evidence-based practice tutorial for nurses: A useful tool and some lessons learned. *The Journal of Continuing Education in Nursing*, *47*(6), 266–271. https://doi.org/10.3928/00220124-20160518-08

Miller, L. C., Graves, R. S., Jones, B. B., & Sievert, M. C. (2010). Beyond Google: Finding and evaluating web-based information for community-based nursing practice. International Journal of Nursing Education Scholarship, *7*, Article31. https://doi.org/10.2202/1548-923X.1961

Neilson, C. J. (2010). Have computers, will travel: Providing on-site library instruction in rural health facilities using a portable computer lab. *Medical Reference Services Quarterly*, *29*(1), 1–9. https://doi.org/10.1080/02763860903484897

Oster, N. H., & Thomas, L. B. (1998). Teaching health information Internet classes in the community: An opportunity for hospital library outreach and collaboration. *Medical Reference Services Quarterly*, *17*(1), 69–74.

Ovaska, T. (2012). Making evidence-based decisions when organising information retrieval training for nurses and head nurses. *Health Information and Libraries Journal*, *29*(3), 252–256. https://doi.org/10.1111/j.1471-1842.2012.00990.x

Owen, D. J. (1997). Using personal reprint management software to teach information management skills for the electronic library. *Medical Reference Services Quarterly*, *16*(4), 29–41.

Pearce-Smith, N., & Hunter, J. (2005). The introduction of librarian tutors into the Teaching Evidence-Based Medicine week in Oxford, UK. *Health Information and Libraries Journal*, *22*(2), 146–149. https://doi.org/10.1111/j.1471-1842.2005.00571.x

Petrak, J., Markulin, H., & Mati, T. (2008). Information literacy in continuing professional development of medical practitioners: A Croatian example. *Health Information & Libraries Journal*, *25*(1), 46–49.

Pettersson, J., Bjorkander, E., Bark, S., Holmgren, D., & Wekell, P. (2017). Using scenario-based training to promote information literacy among on-call consultant pediatricians. *Journal of the Medical Library Association*, *105*(3), 262–267. https://doi.org/10.5195/jmla.2017.79

Pifalo, V. (1994). Outreach to health professionals in a rural area. *Medical Reference Services Quarterly*, *13*(3), 19–26. https://doi.org/10.1300/j115v13n03_02

Purnell, M. (2014). Adding value: Librarian support of a paediatric nurses' journal club. *HLA News*, 7–8.

Ritchie, A. (2011). The Library's role and challenges in implementing an e-learning strategy: A case study from northern Australia. *Health Information and Libraries Journal*, *28*(1), 41–49. https://doi.org/10.1111/j.1471-1842.2010.00923.x

Rosenfeld, P., Salazar-Riera, N., & Vieira, D. (2002). Piloting an information literacy program for staff nurses: Lessons learned. *CIN: Computers, Informatics, Nursing*, *20*(6), 236–243. https://doi.org/10.1097/00024665-200211000-00009

Rothera, H. (2001). From trainees to backers and agents: A programme of electronic information searching skills training for academic staff in health care. *VINE*, *31*(1), 22–28. https://doi.org/10.1108/03055720010803899

Royle, J. A., Blythe, J., Potvin, C., Oolup, P., & Chan, I. M. (1995). Literature search and retrieval in the workplace. *Computers in Nursing*, *13*(1), 25–31.

Schifferdecker, K. E., Reed, V. A., & Homa, K. (2008). A training intervention to improve information management in primary care. *Family Medicine*, *40*(6), 423–432.

Schott, M. (2004). Working for food: Lecturing to physicians. *Journal of Hospital Librarianship*, *4*(3), 15–21.

Schwartz, L. M., & Iobst, B. J. (2008). Magnet again! Librarian's role in research collaboration to maintain Magnet status. *Journal of Hospital Librarianship*, *8*(1), 72–81.

Self, P. C., Sayed, E. N., & Henry, J. K. (1997). "Bridging the Information Gap" for Virginia public health nurses. *Public Health Nursing*, *14*(3), 151–155.

Sinha, L. (2017). Impact of hospital librarian's participation in the Magnet Program: A solo librarian's journey. *Journal of Hospital Librarianship*, *17*(3), 195–200.

Sleutel, M. R., Bullion, J. W., & Sullivan, R. (2018). Tools of the trade: Improving nurses' ability to access and evaluate research. *Journal of Nursing Management*, *26*(2), 167–171. https://doi.org/10.1111/jonm.12529

Smith, S. C., & O'Hagan, E. C. (2014). Taking library instruction into the online environment: One health sciences library's experience. *Journal of the Medical Library Association*, *102*(3), 196–200.

Snowball, R. (2002). 'Find the evidence'—Reflections on an information skills course for community-based clinical health-care staff at the Cairns Library, Oxford. *Health Information & Libraries Journal*, *19*(2), 109.

Sortedahl, C., Wical, S. H., & Benike, J. (2018). Promoting evidence-based practice through a live online nursing journal club: It takes a team. *Journal of Hospital Librarianship*, *18*(1), 15–30.

Steiner, V., Hartmann, J., & Ronau, T. (2002). MedReach: Building an Area Health Education Center medical information outreach system for northwest Ohio. *J Med Libr Assoc*, *90*(3), 317–322.

Stephens, L. C., Selig, C. L., Jones, L. C., & Gaston-Johansson, F. (1992). Research application: Teaching staff nurses to use library search strategies. *Journal of Continuing Education in Nursing*, *23*(1), 24–28.

Stephenson, P. L., Green, B. F., Wallace, R. L., Earl, M. F., Orick, J. T., & Taylor, M. V. (n.d.). Community partnerships for health information training: Medical librarians working with health-care professionals and consumers in Tennessee. *Health Information & Libraries Journal*, *21*, 20–26.

Thompson, H. (2018). Managing a biomedical library's instruction program: Redefining scope. *Medical Reference Services Quarterly*, *37*(1), 97–104.

Twose, C., Swartz, P., Bunker, E, Roderer, N. K., & Oliver, K. B. (2008). Public health practitioners' information access and use patterns in the Maryland (USA) public health departments of Anne Arundel and Wicomico Counties. *Health Information & Libraries Journal*, *25*(1), 13–22. https://doi.org/10.1111/j.1471-1842.2007.00738.x

Vela, K., & Bardyn, T. (2019). Increasing rural nurses' awareness of a statewide health information resource: An educational outreach initiative. *Journal of the Medical Library Association*, *107*(2), 244–250. https://doi.org/10.5195/jmla.2019.542

Wallace, R. L. (2007). PDA training of faculty physicians. *Journal of Electronic Resources in Medical Libraries*, *4*(4), 27–39.

Walton, L., Hasson, S., & VanToll Ross, F. A. (2000). Outreach to public health professionals: Lessons learned from a collaborative Iowa public health project. *Bulletin of the Medical Library Association*, *88*(2), 165–171.

Watson, M. M., Timm, D. F., Parker, D. M., Adams, M., Anderson, A. D., Pernotto, D. A., & Comegys, M. (2006). Using a portable wireless computer lab to provide outreach training to public health workers. *Medical Reference Services Quarterly*, *25*(4), 1–9. https://doi.org/10.1300/J115v25n04_01

Wender, R. W., & Thompson, C. M. (1989). Teaching MEDLINE to non-urban end users. *Medical Reference Services Quarterly*, *8*(2), 25–40.

Winsett, R. P., & Moutseous, M. (2012). Collaborating with hospital librarians to engage nurses in evidence-based practice education. *Journal of Hospital Librarianship*, *12*(4), 309–316.

Yeoh, J. (1991). Librarian or educator: Into the classroom. *Nurse Education Today*, *11*(1), 70–73. https://doi.org/10.1016/0260-6917(91)90128-w
